# Supplementary material for: Phylogenetic Marker Selection and Protein Sequence Analysis of the ORF5 Gene Product of Grapevine Virus A
Source: Plants (Basel). 2022 Apr 20;11(9):1118. doi: 10.3390/plants11091118 (PMC9104223; doi:10.3390/plants11091118)
Supplement: Supplementary file 1 [file plants-11-01118-s001.zip › supplementary tables.pdf]

plants

MDPI

Supplementary Table S1. Accession numbers, hosts and origins of Grapevine virus A isolates/strains used in this study for partial ORF5 analysis.

Isolate	Accession no.	Host	origin
T6 -Torkaman	MG551301	*Vitis vinifera*	Iran
UC -Urmia	MG551302	*Vitis vinifera*	Iran
VJ8 -Varjoy	MG551303	*Vitis vinifera*	Iran
UN1 -Nazloo	MG551304	*Vitis vinifera*	Iran
CYL3-siloo	MG551305	*Vitis vinifera*	Iran
MR Maragheh	MG551306	*Vitis vinifera*	Iran
BEN1 khaneh beig	MG551307	*Vitis vinifera*	Iran
NR Narjabad	MG551308	*Vitis vinifera*	Iran
IR-S7	GU084163	*Vitis vinifera*	Iran
IR-G1	GU084164	*Vitis vinifera*	Iran
IR-BV	GU084167	*Vitis vinifera*	Iran
GVD	Y07764	*Nicotiana occidentalis*	Italy
Is 151	X75433	*Nicotiana benthamiana*	Italy
isolate="1-19-1-1	HQ671645	*Vitis vinifera* x *Vitis labrusca*	China
isolate 1-21-5-1	HQ671650	*Vitis vinifera* x *Vitis labrusca*	China
isolate 2-9-7-9	HQ671657	*Vitis vinifera* x *Vitis labrusca*	China
isolate 12-3-7-4	HQ671638	*Vitis vinifera* x *Vitis labrusca*	China
4-7-9-8	HQ671666	*Vitis vinifera* x *Vitis labrusca*	China
8-21-3-3	HQ671644	*Vitis vinifera* x *Vitis labrusca*	China
12-3-7-6	HQ671628	*Vitis vinifera* x *Vitis labrusca*	China
9-29-9-1	HQ671641	*Vitis vinifera* x *Vitis labrusca*	China
10-29-5-5	HQ671635	*Vitis vinifera* x *Vitis labrusca*	China
10-26-4-2	HQ671632	*Vitis vinifera* x *Vitis labrusca*	China
2-3-6-6	HQ671653	*Vitis vinifera* x *Vitis labrusca*	China
2-4-4-7	HQ671648	*Vitis vinifera* x *Vitis labrusca*	China
9-29-8-2	HQ671640	*Vitis vinifera* x *Vitis labrusca*	China
3-30-9-1	HQ671663	*Vitis vinifera* x *Vitis labrusca*	China
1-21-5-3	HQ671651	*Vitis vinifera* x *Vitis labrusca*	China

1-27-8-5	HQ671659	*Vitis vinifera* L. x *Vitis labrusca*	China
1-26-7-5	HQ671655	*Vitis vinifera* L. x *Vitis labrusca*	China
3-3-7-3	HQ671658	*Vitis vinifera* x *Vitis labrusca*	China
3-4-4-4	HQ671627	*Vitis vinifera* x *Vitis labrusca*	China
3-10-5-8	HQ671652	*Vitis vinifera* x *Vitis labrusca*	China
3-16-8-5	HQ671661	*Vitis vinifera* x *Vitis labrusca*	China
4-2-9-3	HQ671665	*Vitis vinifera* x *Vitis labrusca*	China
GTR1SD-1	DQ855081	*Vitis vinifera* cv. Shiraz	South Africa
P163-M5	DQ855082	*Vitis vinifera* cv. Cinsaut Blanc	South Africa
KWVMo4-1	DQ855083	*Vitis vinifera* cv. Merlot	South Africa
BMo32-1	DQ855087	*Vitis vinifera* cv. Merlot	South Africa
JP98	AF441235	*Nicotiana benthamiana*	South Africa
GTR1-2	DQ855086	*Vitis vinifera* cv. Shiraz	South Africa
92/778	AF441234	*Nicotiana benthamiana*	South Africa
GTG11-1	DQ855084	*Vitis vinifera* cv. Shiraz	South Africa
MSH18-1	DQ855085	*Vitis vinifera* cv. Shiraz	South Africa
I327-5	KC962564	*Vitis vinifera* cv. Shiraz	South Africa
GVA	AY244516	*Nicotiana benthamiana*	South Africa
isolate 3	KF594433	*Vitis vinifera* Frankovka variety	Macedonia
4	KF594434	*Vitis vinifera* Vranec variety	Macedonia
2	KF594432	*Vitis vinifera* Vranec variety	Macedonia
TRAJ2-BR	KX828703	*Vitis vinifera* cv. Trajadura	Brazil
PA3	AF007415	*Nicotiana benthamiana*	Israel
32PA	KF029735	*Vitis vinifera*	Poland
7PA	KF029733	*Vitis vinifera*	Poland
23PA	KF029734	*Vitis vinifera*	Poland
92PA	KF029736	*Vitis vinifera*	Poland
IH11	JN565032	*Vitis vinifera*	Poland
IH8	JN565031	*Vitis vinifera*	Poland

Supplementary Table S2. Accession numbers, hosts and origins of grapevine virus A isolates/strains used for phylogeny marker analysis in this study.

Isolate	Accession no.	Host	origin
NC_003604_IS151	NC_003604	*Vitis vinifera*	Italy
KC962564_1327_5	KC962564	*Vitis vinifera* cv. Shiraz	South Africa
GTR1-1	DQ787959	*Vitis vinifera* cv. Shiraz	South Africa
GTR1SD-1	DQ855081	*Vitis vinifera* cv. Shiraz	South Africa
P163-M5	DQ855082	*Vitis vinifera* cv. Cinsaut Blanc	South Africa
KWVMo4-1	DQ855083	*Vitis vinifera* cv. Merlot	South Africa
GTR1-2	DQ855086	*Vitis vinifera* cv. Shiraz	South Africa
BMo32-1	DQ855087	*Vitis vinifera* cv. Merlot	South Africa
P163-1	DQ855088	*Vitis vinifera* cv. Cinsaut Blanc clone P163/12	South Africa
TRAJ2-BR	KX828703	*Vitis vinifera* cv. Trajadura	Brazil
VB-108	MF979533	*Vitis vinifera* cv. Babica	Croatia
P70	MG925333	*Vitis vinifera* cv. Pinot Noir	France
TT2017_74_47	MK404720	*Vitis vinifera* cv. Pinot Noir	France
TT2017_74_53	MK404721	*Vitis vinifera* cv. Pinot Noir	France
TT2017_79	MK404722	*Vitis vinifera* cv. Pinot Noir	France
PA3	AF007415	*Vitis vinifera*	Israel
3138-03	JX559641	*Vitis vinifera*	Canada
GVD-MD25	MF774336	*Vitis vinifera* cv. Malvasia Dubrovacka	USA
